# Digital eye strain and its associated factors among radiology physicians in Pakistan: a cross-sectional survey using logistic regression analysis

**DOI:** 10.1097/MS9.0000000000001882

**Published:** 2024-03-04

**Authors:** Muhammad Junaid Tahir, Ummey Aymen, Qasim Mehmood, Muhammad Sohaib Asghar, Usha Kumari, Zair Hassan, Nasreen Naz, Nida Rafiq, Muhammad Tayyeb, Irfan Ullah, Khabab Abbasher Hussien Mohamed Ahmed, Osamah Alwalid

**Affiliations:** a Lahore General Hospital; b King Edward Medical University, Lahore; c Dow University of Health Sciences, Karachi; d Lady Reading Hospital; e Kabir Medical College, Gandhara University, Peshawar; f Bacha Khan Medical College, Mardan, Pakistan; g Mayo Clinic-Rochester, MN; h Faculty of Medicine, University of Khartoum, Khartoum, Sudan; i Department of Diagnostic Imaging, Sidra Medicine, Doha, Qatar

**Keywords:** computer vision syndrome, radiographs, radiologyscreen, teleradiology, visual disorder

## Abstract

**Background and aims:**

Increased use of digital devices in the modern era has led to the development of digital eye strain (DES) or computer vision syndrome in their users. This can result in the development of various ocular and visual symptoms among them. In this study, the authors aimed to view the prevalence of digital eye strain among radiology physicians in Pakistan and their associated risk factors.

**Materials and methods:**

A cross-sectional study was conducted to evaluate occupational DES among radiology physicians in Pakistan. The data collection was done using the convenience sampling technique, and the data were analyzed using IBM SPSS for Windows, Version 25.0.

**Results:**

Out of the 247 respondents, 33.6% were males and 66.4% were females. 41.7% of them were between 30 and 40 years of age and 51.8% of them were radiology residents. 52.2% of the participants had a refractive error and were using a corrective lens. The majority of the radiologists in our study (84.2%) preferred picture archiving and communication system (PACS) over films and 82.2% of them reported having breaks of less than 15 min. Major symptoms reported by the participants were tired or heavy eyes (69.6%) and headache (69.3%). The proportion of developing DES was higher in females [*P*=0.001, adjusted odds ratio (aOR)=2.94], radiology residents (*P*=0.031, aOR=3.29), and working hours of more than 4 h per day (*P*<0.001, aOR=0.04).

**Conclusion:**

With recent advances in the field of radiology in Pakistan, the frequency of developing DES among radiologists is increasing. Being a female, having long working hours, and having noticeable flickers on the digital screens were among the significant factors in developing DES among radiologists.

## Introduction

Highlights

- Digital eye strain (DES) or computer vision syndrome is a condition that has been linked to the increased use of digital gadgets in the modern day. Various ocular and visual problems may arise as a consequence of this.
- The incidence of developing DES among radiologists is rising in Pakistan as a result of recent advancements in the industry. We wanted to see how common digital eye strain was among Pakistani radiologists and what risk factors were linked to it.
- To assess occupational depression symptoms (DES) among Pakistani radiology physicians, a cross-sectional study was carried out. Convenience sampling was used to gather the data, and IBM SPSS for Windows, Version 25.0, was used to analyze the results.
- In our study, 84.2% of radiologists said they preferred PACS to films, and 82.2% said they took breaks of less than 15 min. The participants’ primary complaints were headaches (69.3%) and weary or heavy eyes (69.6%). Those who worked more than four hours a day were female, and were residents in radiology had an increased chance of acquiring DES.

As radiology has evolved, radiologists have had to face challenges due to the advancement in the field. One of these is eye strain, to which radiologists are more prone than physicians in other specialities. According to the American Optometric Association, the physical discomfort felt after continuous use of digital screens (computers and other electronic displays) is called digital eye strain (DES) or computer vision syndrome (CVS)^1,2^. Digital devices have become an essential part of our day-to-day lives and are used for various professional and other purposes^3,4^. The prolonged use of these has led to several ocular and visual disorders collectively known as digital eye strain^3^. Digital eye strain is characterized by a complex of ocular and visual symptoms including eye fatigue, eye redness, dry eyes, burning, itching, photophobia, double vision and blurred vision, foreign body sensation, watering or excessive tearing, and headaches^4–6^. Other symptoms include backache due to improper work habits, poor ergonomic design and posture, and neck or shoulder pain that increases with digital screen time^3,7^. The prolonged use of digital devices also increases the risk of developing occupational overuse syndrome characterized by headaches, psychological stress, and injuries to wrists and fingers due to repetitive movements^3^.

Radiology is a field that has evolved histrionically in the last few years and radiologists have to face different challenges due to the advancement going on in this field^8^. Also, radiology is more demanding than other medical specialties as radiologists must view and analyze medical diagnostic images. Since the picture archiving and communication system (PACS) has replaced hard-copy viewing, radiologists must see brightly lit screens for long hours. Furthermore, these advancements have increased the workload of radiologists^4^. Prolonged stress on the ciliary muscles of the eye to adjust the focus on the digital monitors results in ciliary muscle fatigue. Additionally, reduced eye blinking while focusing on the images leads to irritation, dryness, and foreign body sensation in the eyes^9^. Thus, radiologists are the most susceptible among physicians to the development of digital eye strain which leads to a reduced ability to detect minor changes on radiographs^8^. The visual performance of a radiologist is very important for accurate diagnostic interpretations and patient care^4^. This can be viewed both from a short-term approach across a normal working day or a long-term approach across a normal working life. A good radiologist is expected to read the last radiograph of the day with the same accuracy with which he viewed the first radiograph in the morning^10^. Furthermore, the abrupt need for physical distancing in the COVID-19 pandemic has disturbed the workflow of radiologists. Online consultation in the COVID-19 era has increased screen usage among radiologists which could contribute to developing digital eye strain among them^11^.

According to the 2016 Digital Eye Strain Report, the overall prevalence of DES was 65% among 10 000 US adults, with females affected more than males. It was found to be more common in individuals using two or more electronic gadgets at a time^12^. The prevalence was a bit different when recorded for the radiologists in two different studies. It was 50.5% as reported by Dandan *et al.* ^4^. and 36% as reported by Vertinsky *et al.* ^13^. in the radiologists of Saudi Arabia and North America, respectively. Another study conducted on medical students in Pakistan claimed the prevalence of DES-related symptoms among the participants was 67.2%^14^. According to a study done by Anbesu *et al.* ^15^, the pooled prevalence of digital eye strain was 66%, with Pakistan having the highest prevalence and Japan the lowest. However, this area was not well studied among radiologists, who are at the highest risk of developing digital eye strain due to their extended screen time and long working hours. Therefore, this present study aims to view the prevalence of digital eye strain among radiology physicians in Pakistan and their associated risk factors.

## Materials and methods

### Study design and participants

A cross-sectional study was designed to collect data from radiologists using an online Google Docs questionnaire. The survey took 8–10 min to complete, and anonymity was maintained (no personal information was sought or retained).

Along with the survey, participants received a cover letter that explained the aim of the study, informed them of the voluntary nature of participation, and guaranteed their privacy. For any questions about the study, the participants could contact the research investigator (e-mail was provided).

The purpose of this cross-sectional survey was to evaluate occupational digital eye strain among radiology physicians including residents, specialists, and consultants across all public and private hospitals in the major cities (Karachi, Hyderabad, Lahore, Islamabad, Peshawar, Mardan) of Sindh, Punjab, and Khyber Pakhtunkhwa provinces of Pakistan.

### Requirement of participants

The link to the online questionnaire was disseminated among radiologists via WhatsApp, e-mail, and LinkedIn. The personalized link for the survey was shared only among residents, specialists, and consultants of the radiology department, of whom the investigators already have contact information. To ensure that the survey is not compromised by duplicate responses or responses from doctors of any other speciality, each member receiving the link could submit the Google form only once. Two times reminder messages, each after 3 days, were sent to the participants for the completion of the survey.

The data collection was started on 20 September 2021, using the convenience sampling technique, and was open to the participants for 4 weeks, till 18 October 2021. A minimum sample size of 195 participants (using a confidence interval of 95% and a margin of error of 7%) was calculated using online Raosoft sample size calculator. We included only those participants who gave consent and were currently working in the radiology department of their respective hospitals. We excluded interns and observers working in the radiology department. To reach the radiologists whose contact information was not available to the research investigators, a paper-based survey was also distributed among the doctors of the radiology department. Later, completed surveys were collected by the investigators from the respective radiologists.

Overall, 268 responses were collected using both modes (online and paper). The participants who didn’t complete the survey (*n*=12) or didn’t give consent (*n*=9) were excluded from the final sample. Hence, the final sample size was 247 (response rate=92.16%) including 165 responses via an online survey and 82 via a paper-based survey.

### Contents of questionnaire

The authors developed an online survey questionnaire from previously published articles^3,4,6^. The survey comprised a total of 37 questions incorporating the following 5 areas: (1) demographic information, (2) personal eye care, (3) workload-related data, (4) eye strain assessment, 5) workstation design.

### Exposure variables

1. Demographic information: comprised of age, sex, professional ranks (resident, consultant, specialist), current institute of practice (public, private), and year of practice.
2. Personal eye care: including the use of corrective lenses (either glasses or contact lenses) and use of eye drops (Artificial tears, Glaucoma drops, Antibiotic drops) and the number of visits to an ophthalmologist (Never, Within the last year, > 1 year ago).
3. Workload-related data: including duration spent on computer workstation investigating medical images (<4 h, 4–6 h, 7–9 h, >9 h), the duration and type of imaging studies typically reviewed [Screening computed tomography (CT) scan, Diagnostic CT scan, MRI, Sonography, Conventional radiography (plain films), Nuclear medicine studies, Angiography], and frequency of work break taken (once a day, twice a day, every two hours, every hour). The information regarding the mean percentage of time spent on each concern modality was categorized based on time distribution (0%, 1–25%, 26–50%, 51–75%, 76–100%).
4. Eye strain was measured using a set of symptoms that included itching, stinging, or irritated eyes; tired or heavy eyes; difficulty seeing clearly (including blurred or double vision); and headache. On a 5-point Likert scale ranging from “Never”^1^ to “Always,” the prevalence of common eye strain symptoms was assessed^5^.
5. Assessment of radiology included the sort (LCD or CRT), size (e.g. 17 inches, 19 inches, 21 inches, or Screen size varies), and resolution [High resolution (2000 × 2500 pixels), medium resolution (1000 × 1600 pixels), low resolution (512 × 512 pixels), or screen resolution varies]. The height of the workstation and the viewing distance (adjustable vs. nonadjustable) were both questioned by participants. If a participant mentioned having a height-adjustable desk, they were asked if they made their adjustments.

### Outcome variables

The following frequent symptoms were used to assess digital eye strain:

1. Headache.
2. Irritated, burning, or itching eyes.
3. Heavy or tired eyes.
4. Blurred vision.

Senior authors and consultant radiologists confirmed the survey questionnaire’s face and content validity. Each item’s relevancy and appropriateness were discussed. A pilot study with a group of 30 radiologists was then undertaken to examine the clarity of the questions and the time required to complete the survey. Following the pilot research, no substantial adjustments to the questions were made.

The participants’ basic demographic information, workload-related data, and an assessment of digital eye strain symptoms were all addressed by the questionnaire (Supplementary file, Supplemental Digital Content 2, http://links.lww.com/MS9/A396).

After conducting a literature review and conferring with experts, the proposed risk factors were chosen.

### Statistical analysis

Firstly, the information gathered was entered into Microsoft Excel and then imported into the statistical software Statistical Package for Social Sciences (SPSS). The data were analyzed using IBM SPSS for Windows, Version 25.0 software. Basic statistics, such as percentages and frequency distributions of various characteristics, were computed. The χ^2^ test was used to compare categorical variables (or Fischer’s exact test applied if indicated by the limited number of responses). Univariate and multivariate logistic regression analyses were performed, to find out the independent determinants of digital eye strain reporting odds ratio (OR), respective 95% CIs, and statistical power was calculated via Wald’s method. A *p* value less than 0.05 was considered statistically significant.

The work has been reported in accordance with STROCSS guidelines^16^, Supplemental Digital Content 1, http://links.lww.com/MS9/A395.

## Results

Out of the 247 participants, 83 (33.6%) were males and 164 (66.4%) were females. 128 (51.8%) were radiology residents, 68 (27.5%) were consultants and 51 (20.6%) were specialists. 185 (75.9%) of the participants were from public sector institutes and 62 (25.1%) were from private setups. 51 (20.6%) of the study participants never had any eye checkup, 76 (30.8%) of them had their eye examination within the last year and 120 (48.6%) of them had their last eye checkup more than a year ago. (Table 1).

**Table 1:** Baseline characteristics of the study participants (*n*=247)

Variables	Characteristics	Frequency	Percentages
Baselines demographics			
Age	<30 years	89	36.0
	30–40 years	103	41.7
	>40 years	55	22.3
Sex	Male	83	33.6
	Female	164	66.4
Professional rank	Resident	128	51.8
	Consultant	68	27.5
	Specialist	51	20.6
Institution	Public	185	75.9
	Private	62	25.1
Years of practice	<5	143	57.9
	5–10	56	22.7
	>10	48	19.4
Use of corrective lens	No	118	47.8
	Yes	129	52.2
Purpose of wearing corrective lens	For reading	28	11.3
	For distance	37	15.0
	For both	64	25.9
	Not applicable	118	47.8
Use of eye drops	No	234	94.7
	Yes	13	5.3
Last eye examination	Never	51	20.6
	Within last year	76	30.8
	>1 year	120	48.6
Work environment			
Preferred workflow mode	Films	39	15.8
	PACS	208	84.2
Total yearly working time (percent) spent in reviewing cases using films?	0%	32	13.0
	1–25%	112	45.3
	26–50%	64	25.9
	>50%	39	15.8
Total yearly working time (percent) spent in reviewing cases using PACS?	<25%	28	11.3
	26–50%	40	16.2
	51–75%	98	39.7
	>75%	81	32.8
Hours a day (on average) spent reviewing cases?	<4	34	13.8
	4–6	126	51.0
	6–9	70	28.3
	>9	17	6.9
Percentage of time spent working in a week	1–25%	17	6.9
	26–50%	95	38.5
	51–75%	106	42.9
	>75%	29	11.7
The diagnostic technique most frequently used while working	Conventional radiographs (plain films)	29	11.7
	Nuclear medicine studies	2	0.8
	Angiography	4	1.6
	Diagnostic CT	80	32.4
	MRI	16	6.5
	Screening CT (e.g. total-body scans, pulmonary nodule scans)	13	5.3
	Sonography	103	41.7
No. plain radiographs reviewed on average per day	0–25	146	59.1
	26–50	69	27.9
	>50	32	13.0
Cross-sectional imaging studies reviewed on average per day	0–25	149	60.3
	26–50	60	24.3
	>50	38	15.4
Frequency of breaks taken from looking at films	At least every hour	42	17.0
	Every 2 h	60	24.3
	Once a day	101	40.9
	Twice a day	44	17.8
The usual duration of breaks	< 5 min	57	23.1
	5–10 min	90	36.4
	11–15 min	56	22.7
	> 15 min	44	17.8

Data are presented as frequency and percentages.

CT, computed tomography; PACS, picture archiving and communication system.

**Figure 1:**
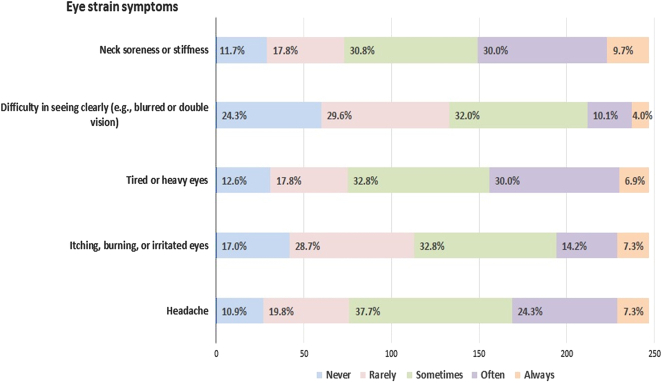
Pattern of reported eye strain symptoms among study participants (*n*=247).

**Figure 2:**
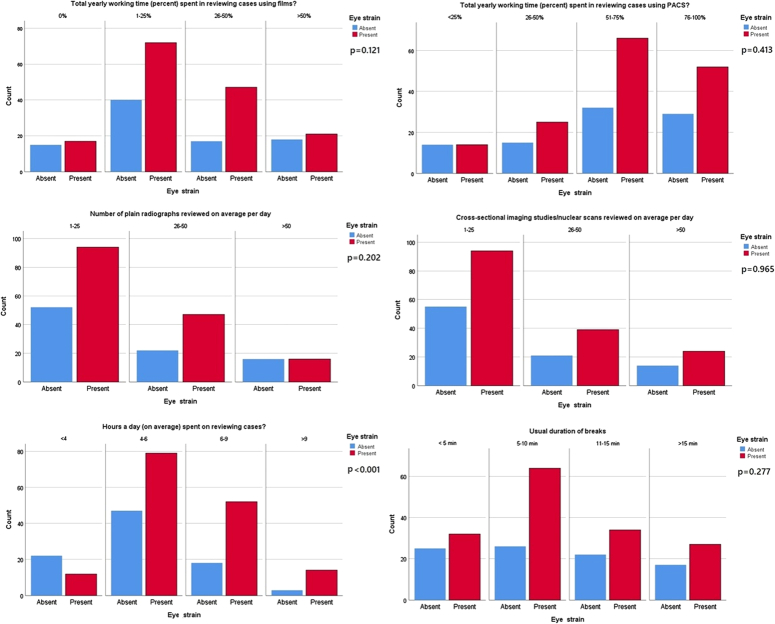
Association of eye strain with work environment (*n*=247). PACS, picture archiving and communication system.

The eye strain symptoms among the radiologist were evaluated by the Likert-like scale as shown in Table 2 and Fig. 1. The frequency of those symptoms indicated by the participants like “always”, “often”, or “sometimes” can be decoded as a “yes”, while “never” or “rarely” is a “no”. 69.3% (*n*=171) of the radiologists reported headache, 54.3% (*n*=134) of them reported irritated eyes and itching or burning in the eyes, while 69.6% (*n*=172) of the study participants reported tired or heavy eyes. Blurred vision or double vision was reported by 46.2% (*n*=114) of the respondents and neck soreness or stiffness was reported by 70.4% (*n*=174) of them. Infodynamics of the workplace were also recorded for the study participants shown in Table 2 and its association with the development of digital eye strain is shown in Fig. 2. The number of monitors used at the work desk of radiologists ranges from one to many. 78.5% (*n*=194) of the study participants indicated liquid crystal display (LCD) as the kind of monitor used in their workstation, 8.1% (*n*=20) of them had cathode ray tube (CRT) and 13.4% (*n*=33) of them had both LCD and CRT in their workstations. 55.5% (*n*=137) of the radiologists indicated their screen size ranges from 7 to 21 inches and 44.5% (*n*=110) of them recorded that their screen size varies. 52.6% (*n*=130) of the respondents reported that their screen resolution ranges from low to high while 47.4% (*n*=117) indicated that their screen resolution varies. 29.6% (*n*=73) of radiologists had a noticeable flicker on their screens.

**Table 2:** Eye strain symptoms

Variables	Characteristics	Frequency	Percentages
Eye strain symptoms			
Headache	Never	27	10.9
	Rarely	49	19.8
	Sometimes	93	37.7
	Often	60	24.3
	Always	18	7.3
Itching, burning, or irritated eyes	Never	42	17.0
	Rarely	71	28.7
	Sometimes	81	32.8
	Often	35	14.2
	Always	18	7.3
Tired or heavy eyes	Never	31	12.6
	Rarely	44	17.8
	Sometimes	81	32.8
	Often	74	30.0
	Always	17	6.9
Difficulty in seeing clearly (e.g. blurred or double vision)	Never	60	24.3
	Rarely	73	29.6
	Sometimes	79	32.0
	Often	25	10.1
	Always	10	4.0
Neck soreness or stiffness	Never	29	11.7
	Rarely	44	17.8
	Sometimes	76	30.8
	Often	74	30.0
	Always	24	9.7
Infodynamics of eyes strain symptoms			
Time of occurrence of most intense symptoms	All day	66	26.7
	Only at the beginning of the day	14	5.7
	Only at the end of the day	167	67.6
Switched to PACS now, but had predominantly used hard-copy films in the past	No	106	42.9
	Yes	141	57.1
Comparison of symptoms with PACS to those when using films	Better now that with PACS use	47	19.0
	About the same with PACS and films	86	34.8
	Slightly worse now with PACS use	71	28.7
	Much worse now with PACS use	43	17.4
Infodynamics of work apparatus			
No. monitors used at the work desk	One	84	34.0
	Two	98	39.7
	More than two	47	19.0
	Number varies	18	7.3
Kind of monitor being used at workstation	LCD (flat screen)	194	78.5
	CRT (Desktop/pc monitor)	20	8.1
	Both	33	13.4
Screen size	7 inches	10	4.0
	19 inches	55	22.3
	21 inches	72	29.1
	Screen size varies	110	44.5
Screen resolution	Low resolution (512 pixels)	9	3.6
	Medium resolution (1000–1600 pixels)	60	24.3
	High resolution (2000–2500 pixels)	61	24.7
	Screen resolution varies	117	47.4
The screen having a noticeable flicker	No	174	70.4
	Yes	73	29.6
Option to optimize the lighting of viewing	No	68	27.5
	Yes	179	72.5
Option to adjust the height of the workstation	No	91	36.8
	Yes	156	63.2
If adjustable, performed height adjustment for work	No	25	10.1
	Yes	131	53.0
	Not applicable	91	36.8
Option to easily adjust viewing distance	No	50	20.2
	Yes	197	79.8

Data are presented as frequency and percentages.

PACS, picture archiving and communication system.

No statistical significance in terms of the prevalence of digital eye strain was observed in terms of most of the demographic variables and work characteristics of the radiologists. However, the proportion of developing digital eye strain was higher in females [*P*=0.001, adjusted odds ratio (aOR)=2.94, 95% CI=1.534–5.664] and radiology residents (*P*=0.031, aOR=3.29, 95% CI=1.117–9.686). Working hours of more than 4 hours per day (*P*<0.001, aOR=0.04, 95% CI=0.008–0.227) were also associated with increased risk. Other factors influencing the prevalence of digital eye strain among radiologists were screens having noticeable flicker (*P*=0.003, aOR=3.30, 95% CI=1.510–7.210) and the option to adjust their viewing height (*P*=0.04, aOR=0.35, 95% CI=0.127–0.972). This is shown in Table 3.

**Table 3:** Regression analysis of study variables with occurrence of eye strain symptoms (*n*=247)

	Univariate analysis			Multivariate analysis		
Study variables	OR	95% CI	*P*	aOR	95% CI	*P*
Sex						
** **Females	1.964	1.141–3.381	0.015*	2.948	1.534–5.664	0.001*
** **Males	Ref	—	—	Ref	—	—
Age						
** **<30 years	1.724	0.863–3.443	0.123	0.824	0.266–2.553	0.738
** **30–40 years	1.551	0.795–3.024	0.198	1.988	0.797–4.956	0.140
** **>40 years	Ref	—	—	Ref	—	—
Profession						
** **Resident	1.942	1.052–3.583	0.034*	3.290	1.117–9.686	0.031*
** **Specialist	0.961	0.463–1.995	0.915	1.082	0.431–2.716	0.867
** **Consultant	Ref	—	—			
Institution						
** **Public	0.946	0.519–1.724	0.857	0.918	0.453–1.856	0.811
** **Private	Ref	—	—	Ref	—	—
Years of practice						
** **<5	1.607	0.818–3.156	0.168	0.872	0.287–2.654	0.810
** **5–10	0.824	0.379–1.794	0.626	0.561	0.202–1.555	0.266
** **>10	Ref	—	—	Ref	—	—
Use of corrective lens						
** **No	Ref	—	—	Ref	—	—
** **Yes	1.234	0.735–2.074	0.427	1.148	0.544–2.419	0.717
Use of eye drops						
** **No	Ref	—	—	Ref	—	—
** **Yes	3.315	0.718–15.305	0.125	3.250	0.569–18.580	0.185
Eye examination						
** **Never	Ref	—	—	Ref	—	—
** **Within last year	1.428	0.685–2.978	0.342	1.428	0.523–3.897	0.487
** **>1 year	1.209	0.619–2.363	0.579	1.508	0.605–3.758	0.378
Preferred technique						
** **Films	0.794	0.395–1.596	0.517	0.944	0.397–2.245	0.897
** **PACS	Ref	—	—	Ref	—	—
Working hours per day						
** **>4	0.117	0.028–0.489	0.003*	0.042	0.008–0.227	<0.001*
** **4–6	0.360	0.098–1.319	0.123	0.188	0.043–0.828	0.027*
** **7–9	0.619	0.159–2.405	0.489	0.445	0.097–2.040	0.297
** **>9	Ref	—	—	Ref	—	—
The screen has a noticeable flicker						
** **No	Ref	—	—	Ref	—	—
** **Yes	2.381	1.280–4.429	0.006*	3.300	1.510–7.210	0.003*
Need to perform height adjustment at the workstation						
** **No	0.368	0.149–0.908	0.030*	0.351	0.127–0.972	0.044*
** **Yes	0.836	0.474–1.474	0.536	0.884	0.452–1.731	0.720
** **Not applicable	Ref	—	—	Ref	—	—

aOR, adjusted odds ratio; OR, odds ratio; PACS, picture archiving and communication system.

* Significant *P* values of less than 0.05. The dependent variable is any of the eye strain symptoms being positive.

## Discussion

Digital eye strain or computer vision syndrome is a group of eye-related problems that is more common in digital screen users^17^. The use of brightly lit screens for more than 2 h significantly increases the incidence of developing digital eye strain^18^. Recent advances in the field of radiology have increased the burden on radiologists as traditional films have been replaced with computerized video display units (VDUs) like PACS^19^. Contemporary radiologists must spend many hours in front of a computer screen due to the recent advent of PACS in the field of radiology^10^. Therefore, radiologists are the most vulnerable among all health workers to the development of this condition^18^. Increased workloads, longer working hours, and fewer breaks between work lead to visual fatigue and tiredness^20^. This further increases the risk and can also lead to errors in diagnostic interpretations^21^.

A total of 247 respondents filled out the survey form; among which 33.6% were males and 66.4% were females. Most of the participants (41.7%) were between 30 and 40 years of age. Most of them (51.8%) were radiology residents and the remaining were consultants and specialists. This is in comparison to another study conducted in Saudi Arabia in which the majority of the participants (40.9%) were radiology residents^4^. Most of the respondents of our study (57.9%) had work experience of fewer than 5 years in the field of radiology.

Most of the participants (52.2%) were having a refractive error and were using a corrective lens; among which only 5.3% were using eye drops for any eye-related symptom. This is in comparison to a study conducted by Dandan *et al.* ^4^, in which 46.5% of the participants were wearing some corrective lenses and 10.1% were using eye drops. 20.6% of the participants of our study never had an eye checkup and 48.6% had an eye checkup more than a year ago. Periodic eye examinations should be recommended among radiologists, especially those of old age to prevent diagnostic errors due to reduced visual acuity^9^.

The majority of the radiologists in our study (84.2%) preferred PACS over films and 59.1% of them reported reviewing fewer than 25 cases per day. 17% of them used to take a break between work after every hour and 24.3% of them used to take breaks every 2 h. This can be correlated to a study conducted in Saudi Arabia in which 11.6% of the participants took a break after every hour and 32.3% of them used to take breaks after every 2 h^4^. The duration of breaks was also asked of the participants. 82.2% of our study participants indicated having breaks of less than 15 min which is in comparison to another study conducted by Dandan *et al.* ^4^, in which 83.9% of the participants had less than 15-min breaks. In another study conducted in Spain, 96% of the participants reported taking less than 15-min breaks between work^6^. Taking frequent breaks between work, no matter what duration can be considered an effective method to prevent digital eye strain among radiologists^8,9^.

Major symptoms reported by the participants were headache (69.3%), itching or burning in the eyes (54.3%), tired or heavy eyes (69.6%), and blurred or double vision (46.2%). This contrasts with another study conducted in Saudi Arabia in which only 26.3% of the participants reported headaches, 21.2% reported itching or burning in the eyes, 29.3% reported tiredness in the eyes, and only 12.1% reported blurred or double vision^4^. In another study conducted by Mohan *et al.* ^5^, 53.9% of the participants indicated itching and headache, and only 11.1% reported blurred or double vision. These findings can also be compared with the findings from a study conducted on medical students of Karachi, in which 38% of the participants reported headaches, 21.8% of them had neck aches, 48% of them reported eye irritation, and 33% of them had burning sensation the eyes^14^.

Lighting in the working room is another factor affecting the development of digital eye strain among radiologists^9^. Most of the participants of our study (72.5%) had an option of adjusting the lighting of their viewing environment, 79.8% of them had an option of adjusting viewing distance, and 63.5% of them had an option of adjusting their viewing height. This is in comparison to another study conducted in Saudi Arabia in which the participants reported that they can adjust the lighting of their work environment (70.2%), viewing distance (64.1%), and viewing height (75.6%)^4^.

Eye strain symptoms were mostly reported by female radiologists (*P*=0.001), radiologists having longer working hours (*P*=0.001) and those having noticeable flicker on their digital screens (*P*=0.003). This is in comparison to the findings of the 2016 Digital Eye Strain Report, according to which digital eye strain is more common among females^12^. The study findings are also in comparison to a study conducted by Ventinky *et al.* ^13^ in which increased eye strain symptoms were reported by female radiologists (*P*<0.001), radiologists having longer workdays (*P*=0.009), and those having noticeable screen flicker on their screens (*P*=0.0003).

Computer Vision Syndrome is highly common in Pakistan and its prevalence was recorded to be highest in Pakistan according to a study conducted by Anbesu *et al.* ^15^. This area was not so studied among the radiologists who are more prone to developing Digital Eye Strain owing to their long screen time. Therefore, we studied its prevalence among the radiologists of Pakistan and its predisposing factors among them.

## Limitations

The present study has some limitations. Digital eye strain symptoms observed in our study were self-reported by the participants, we did not rely on clinical diagnosis since no clinical examination of the participants took place for this purpose. This self-reporting can be subject to bias. More female radiologists filled out the online questionnaire which could have led to a bias in finding an association between sex and developing DES among radiologists. Furthermore, our non-randomized sampling method may influence how representative the sample is and thus cannot rule out selection bias which also limits the generalizability of the results. The study also lacks measures to ensure minimal non-response bias.

## Conclusions

Due to recent advances in the field of radiology, the incidence of developing digital eye strain among radiologists in Pakistan is increasing. Computerized VDUs like PACS have replaced the traditional hard-copy films in radiology. This has led to prolonged working hours in front of digital screens and increased eye strain among radiology physicians. Digital eye strain can decrease the working accuracy of the radiology physicians thus highlighting the need for periodic eye checkups among radiologists. Inadequate light and improper viewing distance in the workplace are also associated with an increased risk of developing this condition. Thus, adequate light and an option to adjust their viewing distance and viewing height at their workplace should be given to all radiology physicians.

## Ethical considerations

The formal ethics approval for the study was taken from “the ethical review board of MTI- Lady Reading Hospital, Peshawar, Pakistan, [712/LRH/MTI]”. The anonymity of the participants was maintained and informed consent was implied for all the participants, whether responding to an online survey or a paper-based survey. This study followed the highest level of ethical standards suggested by the Helsinki Declaration (Revised 2013), and the International Ethical Guidelines for Human Research in Health (2016).

## Consent

Written informed consent was obtained from the patients for publication and any accompanying images. A copy of the written consent is available for review by the Editor-in-Chief of this journal on request.

## Source of funding

There is no funding received for this study.

## Author contribution

M.J.T. and M.S.A. conceived the idea; U.A., U.K., Z.H., N.N., N.R., M.T., and I.U. collected the data; M.S.A. and Q.M. Analyzed and interpreted the data; Q.M., U.A., U.K., N.N., N.R., M.T., M.J.T., and K.A.H.M.A. did write up of the manuscript; and finally O.A., M.S.A., M.J.T., I.U., and Z.H. reviewed the manuscript for intellectual content critically. All authors approved the final version of the manuscript.

## Conflicts of interest disclosure

The author declares no conflicts of interest.

## Research registration unique identifying number (UIN)

1. Name of the registry: Not required.
2. Unique Identifying number or registration ID: Not applicable.
3. Hyperlink to your specific registration (must be publicly accessible and will be checked):

## Guarantor author and authors critical approval

All authors have read and approved the final version of the manuscript have full access to all the data in this study and take complete responsibility for the integrity of the data and the accuracy of the data analysis.

## Data statement and availability

All the relevant study data are available from the corresponding author upon reasonable request.

## Provenance and Peer-review

Not commissioned, externally peer-reviewed.

## Transparency statement

All authors affirm that this manuscript is an honest, accurate, and transparent account of the study being reported; that no important aspects of the study have been omitted; and that any discrepancies from the study as planned (and, if relevant, registered) have been explained.

## Supplementary Material
