# Supplementary material for: Digital eye strain and its associated factors among radiology physicians in Pakistan: a cross-sectional survey using logistic regression analysis
Source: Ann Med Surg (Lond). 2024 Mar 4;86(4):1933–41. doi: 10.1097/MS9.0000000000001882 (PMC10990352; doi:10.1097/MS9.0000000000001882)
Supplement: Supplementary file 2 [file ms9-86-1933-s002.pdf]

# Digital Eye Strain Among Radiologists in Pakistan: A Survey-based Cross-sectional Study

Radiology is an emerging field in modern medical diagnosis and practice and radiologists are at a major risk of developing computer vision syndrome or digital eye strain. Digital eye strain is associated with prolonged use of bright screen devices such as computers, smartphones and tablets. It is suggested that the use of brightly lit devices with unprotected eyes for two consecutive hours is a major risk factor. Therefore, radiologists, with an average screen time of 8 to 9 hours in a workday, are the most vulnerable group for developing digital eye strain among all healthcare professionals.

Participant Consent Form
We invited you to take part in this research study.
We hope you will take part in our study, and to do so, you will be asked to give your consent to proceed. If you decide to take part you are still free to withdraw at any time during or at the end of the questionnaire without giving a reason.

It seeks information regarding some aspects that you eventually face; the responses in this questionnaire will remain confidential and will be used only for research purpose. Your responses will not be in any way a reflection of your job or personality. Your participation in the research will be highly appreciated.

If you have any questions or comments regarding the study please contact.

Thank you and best regards
Email: dr.tjr44@gmail.com

---

*Required

1. I agree to the consent. *

*Mark only one oval.*

- ⬭ Yes
- ⬭ No

## Demographics

2. Age (in years) *

3. Sex *

*Mark only one oval.*

- Female
- Male

4. Professional rank *

*Mark only one oval.*

- Resident
- Specialist
- Consultant

5. Institution *

*Mark only one oval.*

- Public
- Private

6. How many years in practice (including years of training)? *

7. Do you wear corrective lenses (either glasses or contact lenses)? *

*Mark only one oval.*

- Yes
- No

8. If yes, when do you wear them? *

*Mark only one oval.*

- For reading
- For distance
- For both
- Not applicable

9. Do you use eye drops on a regular basis? *

*Mark only one oval.*

- Yes
- No

10. If yes, which kind of drops do you use? *

*Mark only one oval.*

- Artificial tears
- Glaucoma drops
- Antibiotic drops
- Other
- Not applicable

11. When was your last eye examination? *

*Mark only one oval.*

- Never
- Within the last year
- > 1 year ago.

## Working Conditions

12. What technique do you mostly prefer during work: *

*Mark only one oval.*

- Film
- PACS (picture archiving and communication system)

13. If we divide the total yearly working time in 100%, for what percentage of time you review cases using films? *

*Mark only one oval.*

- 0%,
- 1–25%
- 26–50%
- 51–75%
- 76–100%

14. If we divide the total yearly working time in 100%, for what percentage of time you review cases using PACS? *

*Mark only one oval.*

- 0 %
- 1-25%
- 26-50%
- 51-75%
- 76-100%

15. How many hours a day (on average) do you spend reviewing cases? *

*Mark only one oval.*

- <4 hours
- 4–6 hours
- 7–9 hours
- >9 hours

16. Choose Percentage of time in a week you spend in working *

*Mark only one oval.*

- 0%,
- 1–25%,
- 26–50%,
- 51–75%,
- 76–100%

17. Choose the diagnostic technique you mostly use while working. *

*Mark only one oval.*

- Screening CT (e.g., total-body scans, pulmonary nodule scans)
- Diagnostic CT
- MRI
- Sonography
- Conventional radiography (plain films)
- Nuclear medicine studies
- Angiography

18. How many plain radiographs do you review on average per day *

*Mark only one oval.*

- 0–25,
- 26–50,
- 51–75,
- 76–100,
- > 100

19. How many cross-sectional imaging studies (including nuclear medicine scans) do you review on average per day. *

*Mark only one oval.*

- 0–25,
- 26–50,
- 51–75,
- 76–100,
- > 100

20. How often do you take a break from looking at films? *

*Mark only one oval.*

- Once a day
- Twice a day
- Every 2 hours
- At least every hour

21. What is the average duration of a break mentioned above (excluding lunch break)? *

*Mark only one oval.*

- < 5 min
- 5–10 min
- 11–15 min
- > 15 min

Eye–Neck Strain Symptoms

Scale:
1. Never
2. Rarely
3. sometimes
4. often
5. Always

22. How often, from 1 (never) to 5 (always), do you experience Headache during work? *

*Mark only one oval.*

- Never,
- Rarely,
- Sometimes,
- Often,
- Always

23. How often, from 1 (never) to 5 (always), do you experience Itching, burning, or irritated eyes during work? *

*Mark only one oval.*

- Never,
- Rarely,
- Sometimes,
- Often,
- Always

24. How often, from 1 (never) to 5 (always), do you experience tired or heavy eyes during work? *

*Mark only one oval.*

- Never,
- Rarely,
- Sometimes,
- Often,
- Always

25. How often, from 1 (never) to 5 (always), do you experience difficulty in seeing clearly (e.g., blurred or double vision) during work? *

*Mark only one oval.*

- Never,
- Rarely,
- Sometimes,
- Often,
- Always

26. How often, from 1 (never) to 5 (always), do you experience neck soreness or stiffness during work? *

*Mark only one oval.*

- Never,
- Rarely,
- Sometimes,
- Often,
- Always

27. When do you experience these symptoms most intensely? *

*Mark only one oval.*

- Only at the beginning of the day
- Only at the end of the day
- Starting part way through the day and persisting for the rest of the day
- All day

28. If you mostly use PACS now, have you ever used predominantly hard-copy films in the past? *

*Mark only one oval.*

- Yes
- No

29. If yes, then how do the symptoms with PACS compare to those with film? *

*Mark only one oval.*

- Much worse now with PACS use
- Slightly worse now with PACS use
- About the same with PACS and films
- Better now that with PACS use

## Workstation Design (only for those working with PACS)

30. How many monitors do you work with at your workstation? *

*Mark only one oval.*

- One
- Two
- More than two
- Number varies

31. Which kind of monitor do you use? *

*Mark only one oval.*

- LCD (flat screen)
- CRT
- Both
- Don’t know

32. What is the screen size of the monitor you use most of the time? *

*Mark only one oval.*

- 7 inches
- 19 inches
- 21 inches
- Don’t know
- Screen size varies

33. What is the resolution of your monitor? *

*Mark only one oval.*

- High resolution (2,000 × 2,500 pixels)
- Medium resolution (1,000 × 1,600 pixels)
- Low resolution (512 × 512 pixels)
- Don’t know
- Screen resolution varies

34. Does your screen have noticeable flicker? *

*Mark only one oval.*

- Yes
- No

35. Can you optimize the lighting of your viewing environment? *

*Mark only one oval.*

- Yes
- No

36. Is the height of your workstation adjustable? *

*Mark only one oval.*

- Yes
- No

37. If yes, do you ever adjust it? *

*Mark only one oval.*

- Yes
- No
- Not applicable

38. Are you able to easily adjust your viewing distance? *

*Mark only one oval.*

- ( ) Yes
- ( ) No

---

This content is neither created nor endorsed by Google.

Google Forms
